# Gastrointestinal-Sparing Effects of Novel NSAIDs in Rats with Compromised Mucosal Defence

**DOI:** 10.1371/journal.pone.0035196

**Published:** 2012-04-09

**Authors:** Rory Blackler, Stephanie Syer, Manlio Bolla, Ennio Ongini, John L. Wallace

**Affiliations:** 1 Farncombe Family Digestive Health Research Institute, McMaster University, Hamilton, Ontario, Canada; 2 NicOx Research Institute, Milan, Italy; University of Aberdeen, United Kingdom

## Abstract

Nonsteroidal anti-inflammatory drugs are among the most commonly used prescription and over-the-counter medications, but they often produce significant gastrointestinal ulceration and bleeding, particularly in elderly patients and patients with certain co-morbidities. Novel anti-inflammatory drugs are seldom tested in animal models that mimic the high risk human users, leading to an underestimate of the true toxicity of the drugs. In the present study we examined the effects of two novel NSAIDs and two commonly used NSAIDs in models in which mucosal defence was expected to be impaired. Naproxen, celecoxib, ATB-346 (a hydrogen sulfide- and naproxen-releasing compound) and NCX 429 (a nitric oxide- and naproxen-releasing compound) were evaluated in healthy, arthritic, obese, and hypertensive rats and in rats of advanced age (19 months) and rats co-administered low-dose aspirin and/or omeprazole. In all models except hypertension, greater gastric and/or intestinal damage was observed when naproxen was administered in these models than in healthy rats. Celecoxib-induced damage was significantly increased when co-administered with low-dose aspirin and/or omeprazole. In contrast, ATB-346 and NCX 429, when tested at doses that were as effective as naproxen and celecoxib in reducing inflammation and inhibiting cyclooxygenase activity, did not produce significant gastric or intestinal damage in any of the models. These results demonstrate that animal models of human co-morbidities display the same increased susceptibility to NSAID-induced gastrointestinal damage as observed in humans. Moreover, two novel NSAIDs that release mediators of mucosal defence (hydrogen sulfide and nitric oxide) do not induce significant gastrointestinal damage in these models of impaired mucosal defence.

## Introduction

The ability of nonsteroidal anti-inflammatory drugs (NSAIDs) to cause significant ulceration and bleeding in the stomach and duodenum is well recognized [1]. Less appreciated, until the recent advent of video capsule endoscopy, is the full extent of detrimental effects these drugs exert on the intestinal tract distal to the ligament of Treitz [2], which appear to be produced via mechanisms distinct from those responsible for gastro-duodenal injury [1], [3]. Therapies aimed at preventing NSAID-induced gastrointestinal (GI) injury have largely focused on gastroduodenal damage. The most common approach used clinically to minimize gastroduodenal injury is to co-administer a proton pump inhibitor (PPI) with the NSAID. This has been shown to significantly reduce the incidence of gastro-duodenal damage [4], but recent animal studies suggest that suppression of acid secretion can lead to exacerbation of NSAID-induced small intestinal injury and bleeding [5]. There are several clinical studies that report high levels of intestinal damage in healthy volunteers taking NSAIDs plus a PPI [6]–[9], and one study showing significant elevation of a marker of intestinal inflammation (calprotectin) in patients taking PPIs [10].

Selective inhibitors of cyclooxygenase (COX) -2 entered the marketplace at the turn of the last century with great promise for GI safety. This promise has largely been unfulfilled [11]. However, even the small upper GI benefit gained through use of a selective COX-2 inhibitor versus a non-selective COX inhibitor is lost when low-dose aspirin is co-administered [12], [13]. This co-therapy is aimed at reducing the incidence of cardiovascular events associated with the use of selective and most non-selective NSAIDs [14]. Low-dose aspirin, alone, can also cause significant small intestinal injury [15]. Studies to evaluate the effects on the GI tract of the combined use of an NSAID, a PPI and low-dose aspirin, which is now a common combination in clinical practice, have not been reported.

One of the problems encountered in attempts to develop GI-sparing NSAIDs is that preclinical studies have largely focused on the stomach (ignoring the small intestine) and are usually performed using healthy animals. The latter may give false security about the safety of the drug, which in humans will be used by individuals with significant co-morbidities and compromised mucosal defence. It is therefore important to evaluate the safety and efficacy of novel NSAIDs in models that more closely resemble the patients who will be the major users of these drugs. NSAID-induced gastroduodenal injury has been reported to be elevated in elderly patients, and in patients with co-morbidities such as obesity, hypertension and rheumatoid arthritis [16]–[18]. Novel NSAIDs should also be evaluated in combination with the drugs that are often co-prescribed with NSAIDs (e.g., proton pump inhibitors and low-dose aspirin), given that these drugs may exacerbate NSAID-induced GI damage. This approach will make the data more predictive of the human response, therefore providing more insight on the potential GI safety of drugs intended for use as treatments of inflammatory conditions.

In the present study, we examined the effects of a number of NSAIDs in models that attempt to mimic relevant clinical scenarios of NSAID use. Two of the most commonly used NSAIDs (naproxen and celecoxib) were compared to each other and to two novel, putative GI-sparing NSAIDs (both chemically related to naproxen; one nitric oxide-releasing and the other hydrogen sulfide-releasing). Both nitric oxide and hydrogen sulfide have been shown to exert protective effects in the GI tract [1], and NSAID compounds that release one of these gaseous mediators produce significantly less GI damage than their respective parent drugs in healthy animals [19], [20].

In addition to examining the GI safety of these compounds when administered together with low-dose aspirin and/or a PPI, we evaluated them in models in which mucosal defence may be compromised (i.e., obese rats, arthritic rats, hypertensive rats and aged rats). In all studies we compared the test drugs at doses that produced comparable anti-inflammatory effects in rats with adjuvant arthritis.

## Methods

### Animals

Male, Wistar rats weighing 180–220 g and male, Zucker rats (both lean and obese, weighing ∼360 and ∼560 g, respectively), spontaneously hypertensive rats (SHR) and normotensive rats (Wistar-Kyoto; WKR) (180–220 g) were obtained from Charles Rivers (Montreal, QC, Canada). 19-month old, male, Sprague Dawley rats (mean weight of 525 ± 30 g) were obtained from Harlan Laboratories (Indianapolis, IN, USA). All rats were housed in the Central Animal Facility at McMaster University. The rats were fed standard chow and water *ad libitum*, and were housed in pairs in a room with controlled temperature (22 ± 1°C), humidity (65–70%) and light cycle (12 h light/12 h dark). All experimental procedures described herein were approved by the Animal Care Committee of the Faculty of Health Sciences at McMaster University. The studies were carried out in accordance with the guidelines of the Canadian Council of Animal Care. The health of the animals was assessed at least twice-daily, and any animals in distress or having lost >15% of their original body weight were euthanized by an overdose of sodium pentobarbital.

### Test Drugs

Naproxen and ATB-346 (2-(6-methoxy-napthalen-2-yl)-propionic acid 4-thiocarbamoyl-phenyl ester) were tested in all models, and in some models the effects of celecoxib and NCX 429 [(S)-6-(nitrooxy)hexyl 2-(6-methoxynaphthalen-2-yl)propanoate] were also examined. Naproxen and celecoxib were administered at a dose of 10 mg/kg. This dose was selected because it produced significant and comparable activity in reducing paw swelling in rats with adjuvant arthritis (see below) [21]. In all studies described below, ATB-346 and NCX 429 were given at doses equimolar to the dose of naproxen. All test drugs were suspended in dimethylsulfoxide/1% carboxymethylcellulose; 5∶95 ratio).

### Adjuvant Arthritis Model

Polyarthritis was induced in Wistar rats via an injection into the base of the tail of 100 µL of Freund’s Complete Adjuvant containing 0.75 mg of heat-killed *Mycobacterium butirricum* [21]. The volume of the hindpaws of each rat was blindly measured using a hydroplethysmometer (Ugo Basile, Comerio, Italy) prior to the injection of the adjuvant, and on days 7, 10, 14 and 18 after adjuvant administration. Groups of rats (n = 8 each) were treated twice-daily beginning on day 7 with celecoxib (10 mg/kg), naproxen (10 mg/kg), or equimolar doses of ATB-346 (14.5 mg/kg) or NCX 429 (15 mg/kg). Two control groups (one with adjuvant arthritis and one naive) were treated with an equal volume of vehicle. At the end of the study the stomach and small intestine were excised and blindly evaluated for hemorrhagic damage, as described below.

### NSAID-Induced Gastroenteropathy

Unless otherwise noted, studies of NSAID-induced gastroenteropathy were performed in healthy (2-month old) Wistar rats. Rats were given one of the test drugs or vehicle orally, twice each day for 4.5 days (9 administrations in total). Three hours after the final administration of drug or vehicle, the rats were anesthetized with sodium pentobarbital and blood was drawn from the aorta for ELISA measurement of whole blood thromboxane B_2_ (TXB_2_)_­_ synthesis, as an index of systemic COX-1 activity [22]. The stomach and small intestine were then blindly evaluated for hemorrhagic damage. This involved measuring the lengths, in mm, of all hemorrhagic lesions. Separate gastric and intestinal damage scores were then calculated by summing the lengths of all lesions for each rat [5]. After scoring, samples of the jejunum and of the corpus region of the stomach were collected for the measurement of prostaglandin (PG)E_2_ synthesis, as described previously [23]. Finally, specimens of gastric and jejunal tissues were fixed and processed for histological examination (H&E staining).

### Polypharmacy Model

Groups of Wistar rats (n>6/group) were treated for a total of 9 days with one or more drugs. The rats received omeprazole (10 mg/kg) or vehicle twice-daily (ip) throughout the 9 days. Beginning on day 2, the rats received vehicle or low-dose aspirin (10 mg/kg) orally once daily. Beginning on day 5, the rats received an NSAID or vehicle orally twice-daily. The rats were euthanized 3 hours after the final administration of the NSAID or vehicle for blind evaluation of the extent of damage to the stomach and small intestine, as described above. Samples were taken for measurement of prostaglandin and thromboxane synthesis, as described above. Previously we demonstrated that the dose of omeprazole used in this study produced a 99% inhibition of gastric acid secretion by the 5^th^ day of administration (when NSAID treatment was initiated) [5]. Daily administration of aspirin at 10 mg/kg produced 95% inhibition of whole blood thromboxane synthesis by the 3^rd^ day of administration (when NSAID treatment was initiated) [5].

### Advancing Age Model

Studies were performed, as described above, using rats that were 19 months of age (n = 6/group).

### Obesity Model

Male, Zucker rats of the fa/fa phenotype spontaneously develop to an obese state, whereas their Fa/fa littermates exhibit normal weight gain [24]. Obese and lean Zucker rats (n = 6/group) were treated orally twice-daily with naproxen (10 mg/kg), celecoxib (10 mg/kg), ATB-346 (14.5 mg/kg), or vehicle (1% CMC, DMSO (95∶5)) for a total of 4.5 days. 3 hours after the final dose, the rats were euthanized, the stomach and small intestine were blindly evaluated for damage and sample collection was conducted as described above (*NSAID-Induced Gastroenteropathy*). In order to determine if any of the rats were diabetic, as has been reported [25], blood glucose levels were determined prior to and after NSAID dosing using a Freestyle Freedom Lite unit (Abbott Diabetes Care, Saint-Laurent, QC, Canada).

### Hypertension Model

To confirm that the spontaneously hypertensive rats were indeed hypertensive, blood pressure was measured in SHR and normotensive WKR using a CODA Non-Invasive (tail-cuff) Blood Pressure System (Kent Scientific Corporation, Torrington, CT, USA). In order to minimize stress-induced alterations in blood pressure, each rat underwent a daily 15-minute training session in the device for 3 days prior to blood pressure determination. The rats were acclimated for 10 min in advance of blood pressure readings and placed on a heating blanket (36°C) to promote thermo-regulation and maintain tail blood flow. Blood pressure measurements were performed two days prior to beginning NSAID administration. The rats received naproxen (10 mg/kg), an equimolar dose of ATB-346 (14.5 mg/kg) or vehicle orally twice-daily for 4.5 days. 3 hours after the final administration of the test drugs the rats were euthanized and the extent of gastric and small intestinal damage was blindly evaluated, as described above. Samples were taken for measurement of gastric PGE_2_ and whole blood TXB_2_ synthesis, as described above.

### Pharmacokinetics

Rats were treated with a single, oral dose of naproxen (10 mg/kg), ATB-346 (14.5 mg/kg) or NCX 429 (15 mg/kg). Subgroups (n = 4 each) of rats were anesthetized with sodium pentobarbital 4 h later. The bile duct was cannulated and bile was collected for 30 minutes [26], after which a blood sample was drawn from the descending aorta. 50 µL of bile or serum samples were de-proteinated by adding three volume of acetonitrile and 10 µL of dimethylsulfoxide. Samples were then centrifuged for 10 min at 4°C (3200×*g*) and the supernatants were transferred to a 96-well plate for analysis using a LC-MS/MS system for quantification of naproxen [5]. A calibration curve of naproxen in both serum and bile was prepared in the concentration range 0.1–300 µM.

### Materials

ATB-346 (2-(6-methoxy-napthalen-2-yl)-propionic acid 4-thiocarbamoyl-phenyl ester) was provided by Antibe Therapeutics Inc. (Toronto, ON, Canada) and NCX 429 [(S)-6-(nitrooxy)hexyl 2-(6-methoxynaphthalen-2-yl)propanoate] was provided by NicOx S.A. (Sophia Antipolis, France). Celecoxib was obtained from American Custom Chemical Corp. (San Diego, CA, USA). Sodium naproxen and streptozotocin were purchased from Sigma-Aldrich (St. Louis, MO, USA). ELISA kits for measuring TXB_2_and PGE_2_were obtained from Cayman Chemicals (Ann Arbor, MI, USA). Freund’s Complete Adjuvant and *Mycobacterium butirricum* were purchased from Difco Laboratories (Detroit, MI, USA).

### Statistical Analyses

Data are expressed as the mean ± SEM. Comparisons among groups of data were performed by one-way analysis of variance followed by a post hoc test (Dunnett’s Multiple Comparison Test for parametric data and Mann Whitney Test for non-parametric data). An associated probability (p value) of less than 5% was considered significant.

## Results

### Efficacy Studies

**Figure 1:**
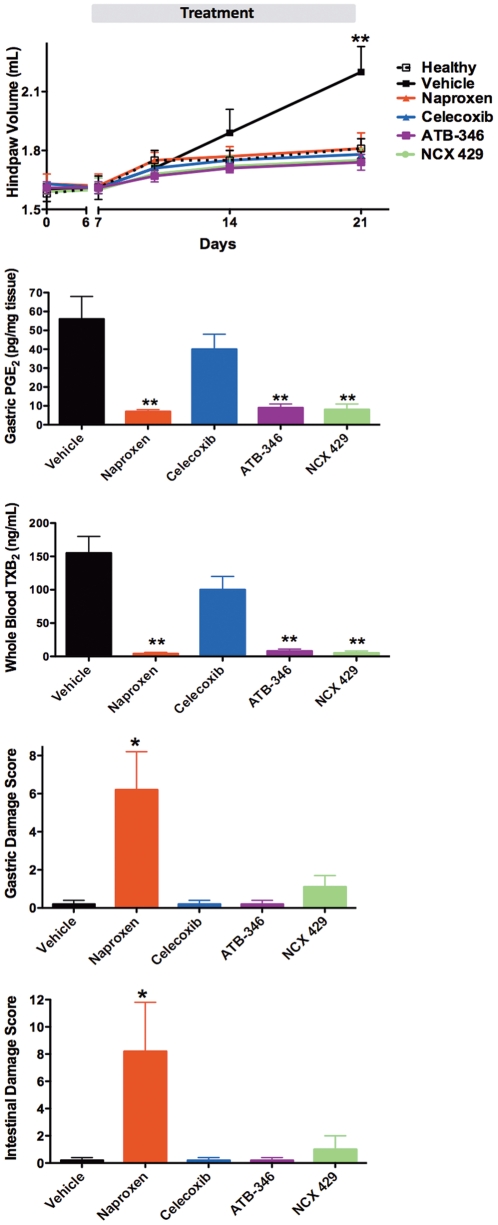
Effects of NSAIDs in rats with adjuvant arthritis. Despite comparable suppression of paw swelling (panel A), gastric prostaglandin synthesis (panel B) and whole blood thromboxane synthesis (panel C), ATB-346 and NCX 429 did not cause significant gastric (panel D) or intestinal (panel E) damage. Celecoxib also did not cause significant GI damage. *p<0.05, **p<0.01 versus the naproxen-treated group. n = 8 per group.

Rats with adjuvant arthritis were treated from day 7 to day 21, twice-daily, with the test drugs or vehicle. Fig. 1A illustrates that each of the drugs, at the doses tested, produced comparable reductions in paw edema, reducing paw volumes to levels not different from those of healthy rats. Consistent with the comparable anti-inflammatory effects, the test drugs produced comparable suppression of gastric prostaglandin E_2_ synthesis (Fig. 1B) and systemic COX-1 activity (whole blood thromboxane synthesis) (Fig. 1C). Despite this, the extent of gastric and small intestinal damage differed among the treatment groups. Naproxen caused significant gastric and intestinal damage, while the levels of damage were negligible with celecoxib, ATB-346 and NCX 429 (not significantly different from that in vehicle-treated rats) (Fig. 1D and 1E).

### GI Damage in Healthy, Young Rats

**Figure 2:**
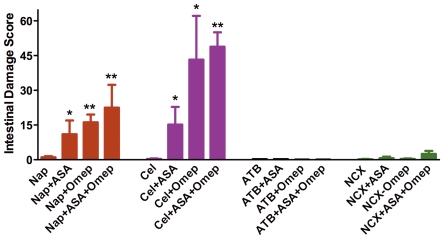
Co-administration of naproxen or celecoxib with omeprazole and/or low-dose aspirin results in marked exacerbation of small intestinal damage. In contrast, rats given a naproxen derivative (ATB-346 or NCX 429) did not develop significant intestinal injury when given alone or in combination with omeprazole, low-dose aspirin, or both. *p<0.05, **p<0.01 versus the corresponding group treated with NSAID alone (n≥6 per group). Aspirin and omeprazole, alone or given together, did not elicit significant intestinal damage.

In healthy, 2-month old Wistar rats, administration of naproxen twice-daily for 4.5 days results in very little detectable damage in the stomach or small intestine. The mean damage scores were 1.3 ± 0.5 for the stomach and 1.0 ± 0.5 for the small intestine (n =  8; see Fig. 2 for intestinal damage). Similarly, celecoxib, ATB-346, NCX 429 and low-dose aspirin produced negligible gastric and small intestinal damage in healthy young rats.

### Polypharmacy Model

The test drugs were also assessed for their ability to induce gastrointestinal damage in circumstances mimicking the clinical scenario in which patients receive co-treatment with a ‘gastroprotective’ drug and/or low-dose aspirin (to provide ‘cardio-protection’). Gastric damage remained low in rats co-treated with one of the NSAIDs and omeprazole and/or low-dose aspirin (mean damage scores of <3). However, when naproxen or celecoxib were administered together with low-dose aspirin, the extent of hemorrhagic injury in the small intestine increased markedly (p<0.05) over that observed with either drug alone (Fig. 2). Similarly, co-administration of the proton pump inhibitor (omeprazole) with naproxen or celecoxib resulted in a dramatic increase in the extent of small intestinal damage. The combination of either naproxen or celecoxib with both low-dose aspirin and omeprazole resulted in the highest intestinal damage scores (Fig. 2).

In sharp contrast to the effects observed with naproxen and celecoxib, administration of ATB-346 or NCX 429 together with low-dose aspirin and/or omeprazole did not result in significant intestinal damage (Fig. 2).

### Studies in Aged Rats

**Figure 3:**
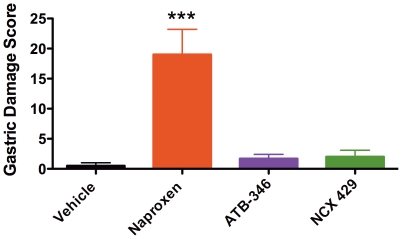
Older (19 months of age) rats develop extensive gastric damage when given naproxen, but not when given equimolar doses of a hydrogen sulfide-releasing naproxen derivative (ATB-346) or a nitric oxide-releasing naproxen derivative (NCX 429). ***p<0.001 versus the vehicle-treated group. n =  6 rats per group.

In rats that were 19 months of age, twice-daily administration of naproxen for 4.5 days resulted in the development of extensive gastric damage that consisted of both of erosions and penetrating ulcers (Fig. 3). Histological evaluation confirmed that damage in the older rats penetrated through the muscularis mucosae into the submucosa. In contrast to the studies in younger rats, the older rats did not develop significant intestinal damage when treated with naproxen. Older rats treated with celecoxib, ATB-346 or NCX 429 did not develop significant gastric or intestinal damage. However, ATB-346 and NCX 429 suppressed (>95%) gastric PGE_2_ and whole blood thromboxane synthesis to the same extent as naproxen.

### Studies in Obese Rats

**Figure 4:**
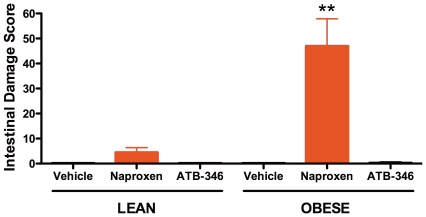
Increased naproxen-induced small intestinal damage in obese versus lean rats. Neither lean nor obese rats developed intestinal damage when given ATB-346. **p<0.01 versus the corresponding vehicle- and ATB-346-treated rats. n = 6 rats per group.

Treatment of lean Zucker rats (mean weight of ∼360 g) with naproxen twice-daily for 4.5 days resulted in a small amount of damage in the stomach and intestine (Fig. 4), similar to that seen in Wistar rats. Obese Zucker rats (mean weight of ∼560 g) did not exhibit gastric damage, but they developed much more severe small intestinal damage when treated with naproxen (p<0.01 versus the lean counterparts). ATB-346 did not produce detectable gastric or intestinal damage in either lean or obese Zucker rats.

Whole blood thromboxane synthesis was markedly higher in the obese rats than in the lean littermates (632 ± 110 vs 105 ± 36 ng/mL, respectively; p<0.01). However, in both groups of rats naproxen and ATB-346 caused near-complete suppression (>95%) of whole blood thromboxane synthesis and gastric PGE_2_ synthesis.

Diabetes was not observed in any of the Zucker rats. Blood glucose levels were all within the normal range (5.5 ± 1.1 mmol/L in lean vs. 5.5 ± 1.2 mmol/L in obese).

### Studies in Hypertensive Rats

**Figure 5:**
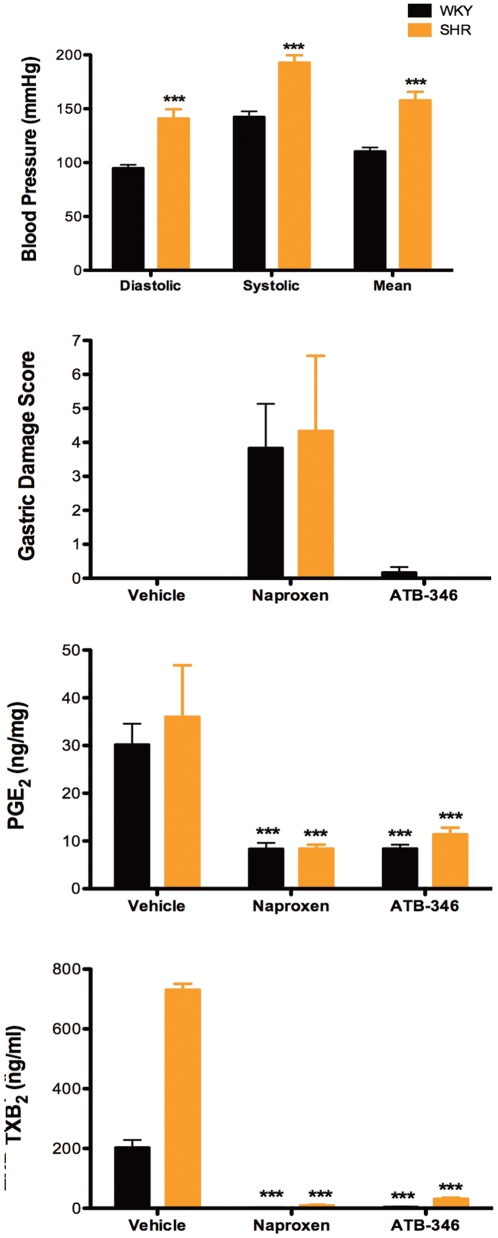
Severity of gastric damage induced by naproxen is similar in spontaneously hypertensive (SHR) and normotensive (WKY) rats. ATB-346 suppressed gastric prostaglandin (PG)E_2_ synthesis and whole blood thromboxane (TXB_2_) synthesis as effectively as naproxen, but did not elicit gastric damage. ***p<0.001 versus the corresponding vehicle-treated group (n = 6 per group).

SHR rats had markedly elevated systemic blood pressure as compared to the normotensive (Wistar-Kyoto) rats (Fig. 5a). When treated with naproxen or ATB-346 twice-daily for 4.5 days, no significant gastric damage was observed in either group of rats (Fig. 5b). Intestinal damage was not observed in either group of rats with either test drug. The test drugs produced comparable suppression of gastric PGE_2_ and whole blood thromboxane synthesis in both hypertensive and normotensive rats (Fig. 5c and 5d). However, the control hypertensive rats did exhibit marked elevations of whole blood thromboxane synthesis (∼3.5-fold), as has been reported previously [27].

### Pharmacokinetics

Four hours after a single dose of naproxen, serum levels of naproxen averaged 98 ± 5 µM. Naproxen levels 4 h after a single dose of ATB-346 were 53 ± µM (not significantly different), while 4 h after a single dose of NCX 429, serum naproxen levels were only 29 ± 8 µM (p<0.05 versus the naproxen-treated group).

When bile levels of naproxen were measured 4 h after administration of the test drugs, some dramatic differences were apparent. In the naproxen-treated group, bile naproxen levels averaged 1.5 ± 0.3 µM, while bile naproxen levels in the rats treated with ATB-346 or NCX 429 were significantly (p<0.05) lower, at 0.5 ± 0.1 and 0.2 ± 0.1 µM, respectively.

**Figure 6:**
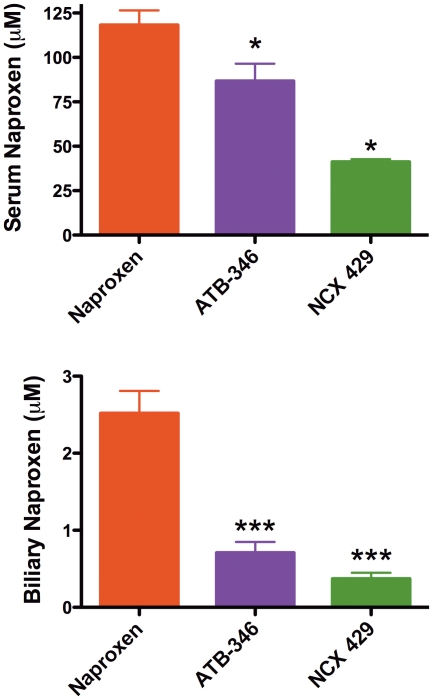
Serum and biliary levels of naproxen are significantly reduced in rats given a naproxen derivative (ATB-346 or NCX 429) as compared to the levels observed in rats given an equimolar dose of naproxen itself. The test drugs were administered twice-daily for 2 days. *p<0.05, **p<0001 versus the naproxen-treated group. n = 4 per group.

Similar differences in serum and bile levels of naproxen were observed in rats treated twice-daily for 2 days with naproxen, ATB-346 or NCX 429 (Fig. 6).

The mass spectrum analysis of bile samples from rats treated once or four times with the test drugs also detected a naproxen glucuronide, which was more evident in the samples from naproxen-treated rats than in samples from rats treated with ATB-346 or NCX 429. Thus, after a single dose of the test drugs, the ratio of the naproxen glucuronide in samples from naproxen-, ATB-346- and NCX 429-treated rats was 22:10:3. After four doses of the test drugs, the ratio changed to 32:9:7.

## Discussion

NSAID-induced gastroenteropathy is a significant limitation to the use of this class of drugs, which is a mainstream therapy for osteoarthritis and other chronic conditions characterized by inflammation and pain. The GI adverse effects of NSAIDs occur more frequently in the elderly, in patients taking anti-platelet/anti-coagulants (including aspirin), and in patients with co-morbidities such as rheumatoid arthritis, obesity, heart failure and hypertension [16]–[18]. Evaluation of tolerability and safety of NSAIDs in animal models that more closely mimic these clinical scenarios will likely provide more relevant predictive data than studies in healthy animals. In the present study, we compared the GI damage of several NSAIDs in rat models of arthritis, polypharmacy, obesity, advancing age and hypertension. Our results demonstrated that in most of these models (hypertension being the exception), the severity of NSAID-induced gastric and/or intestinal damage was markedly elevated as compared to that in healthy rats. In contrast to naproxen (and in some studies celecoxib), hydrogen sulfide- and nitric oxide-releasing naproxen compounds (ATB-346 and NCX 429, respectively) elicited negligible GI damage, despite still inhibiting the key target enzymes for their anti-inflammatory and GI-damaging effects (COX-1 and COX-2). The novel NSAIDs also exhibited comparable anti-inflammatory activity to naproxen and celecoxib in rats with adjuvant arthritis.

Studies in co-morbidity models may provide insights on the pathogenesis of NSAID-gastropathy and –enteropathy. For example, it was interesting that the older rats (19-month old), unlike younger rats (2-month old), developed severe gastric but not intestinal damage. As well as the damage being more extensive in the stomach of older rats, it was also more severe in terms of depth of injury (i.e., penetrating ulcers rather than superficial erosions). The reasons for the propensity of gastric damage (rather than intestinal) in the older rats are unclear, but could be attributable to the reported deficiencies in gastric mucosal defence that occur with age. For example, impaired gastric production of nitric oxide, a key mediator of mucosal defence [28], has been reported in older rats [29], as has reduced gastric prostaglandin synthesis [30].

Arthritic rats exhibited increased susceptibility to gastric and intestinal damage as compared to healthy controls. One of the factors that might contribute to this increase in injury is the enhanced NSAID-induced leukocyte adherence to the vascular endothelium that has been observed in arthritic rats [31]. Leukocyte adherence to the vascular endothelium is a critical early event in pathogenesis of NSAID-gastropathy [32], [33], and also plays an important role in the development of NSAID-induced injury in the small intestine [34].

Bacteria play a role in the initiation and chronicity of ulcers in the intestine and the stomach [35]–[37]. Indeed, NSAID-induced small intestinal damage does not develop in germ-free animals [36]. Obese Zucker rats have a distinct microbiome from their lean counterparts, with a significant reduction of *Bifidobacteria* [38], but we can only speculate, at this point in time, that these differences contribute to the increased susceptibility of obese rats to NSAID-induced enteropathy.

We previously reported that suppression of gastric acid secretion in rats led to a dramatic shift in the microbiota (notably a significant decrease in *Bifidobacteria spp*.) and a marked increase in the susceptibility to NSAID-induced small intestinal damage [5]. The increase in susceptibility to damage could be transferred via the microbiota. In the present study, we extended those finding with the demonstration that administration of low-dose aspirin significantly increases the severity of NSAID-induced small intestinal damage, and found that even greater damage was observed when both omeprazole and low-dose aspirin were co-administered with the NSAID. This is a very common combination of drugs in humans. Proton pump inhibitors are given to reduce the incidence of NSAID-induced gastroduodenal damage, while low-dose aspirin is given to reduce the incidence of NSAID-associated cardiovascular events [12]. No published human studies have examined the effects of co-administration of an NSAID, low-dose aspirin and a proton pump inhibitor on the small intestine. However, a high level of intestinal damage was observed in several video capsule endoscopy studies of healthy, young volunteers given an NSAID plus a PPI over a short period of time [6]–[9], and detrimental effects of low-dose aspirin on the small intestine are well documented [15]. Given the results of the present study, and how widespread the polypharmacy approach is practiced, clinical studies of the GI impact of the combination of an NSAID, low-dose aspirin and a PPI are warranted.

NO and H_2_S have well characterized protective [1], [28], [39], [40] and ulcer-healing [41]–[43] effects in the gastrointestinal tract, and both have been exploited in the design of GI-sparing anti-inflammatory drugs [44], [45]. In the present study, ATB-346 and NCX 429 spared the stomach and small intestine of damage, regardless of the model in which they were tested, and despite the fact that they markedly suppressed mucosal prostaglandin synthesis and platelet thromboxane synthesis. These GI-sparing effects are likely due largely to the mucosal-protective effects of the gaseous mediator released from these drugs (H_2_S from ATB-346 and NO from NCX 429), and to the ability of these mediators to inhibit leukocyte adherence [1], [46]. However, the pharmacokinetic studies suggest another mechanism for the safety of ATB-346 and NCX 429, particularly in terms of tolerability in the small intestine. The enterohepatic circulation of NSAIDs is critical to their ability to induce small intestinal injury [26], [37]. We observed that there were very low levels of naproxen in the bile following administration of ATB-346 or NCX 429, relative to biliary naproxen concentrations following administration of naproxen itself. Unlike naproxen, ATB-346 and NCX 429 do not have free carboxylic acid residues, so would likely have greatly reduced topical irritant properties than naproxen.

In summary, animal models of obesity, advancing age, arthritis and polypharmacy exhibit a significant increase in susceptibility to NSAID-induced GI damage. These models may be more predictive of the properties of NSAIDs in the subset of humans that develop the most adverse GI events when taking these drugs. H_2_S- and NO-releasing derivatives of naproxen were found to be very well tolerated in these co-morbidity models.
